# Clinical efficacy comparison of avapritinib with other tyrosine kinase inhibitors in gastrointestinal stromal tumors with *PDGFRA* D842V mutation: a retrospective analysis of clinical trial and real-world data

**DOI:** 10.1186/s12885-021-08013-1

**Published:** 2021-03-19

**Authors:** Margaret von Mehren, Michael C. Heinrich, Hongliang Shi, Sergio Iannazzo, Raymond Mankoski, Saša Dimitrijević, Gerard Hoehn, Silvia Chiroli, Suzanne George

**Affiliations:** 1 Department of Hematology/Oncology, Fox Chase Cancer Center, 333 Cottman Ave, Philadelphia, PA 19111 USA; 2 Division of Hematology and Medical Oncology, Portland VA Health Care System and Oregon Health & Science University, Knight Cancer Institute, Portland, OR USA; 3 Blueprint Medicines Corporation, Cambridge, MA USA; 4 Dana-Farber Cancer Institute and Harvard Medical School, Boston, MA USA

**Keywords:** GIST, Avapritinib, *PDGFRA* D842V mutation

## Abstract

**Background:**

Avapritinib, a potent inhibitor of KIT and platelet-derived growth factor receptor A (PDGFRA) tyrosine kinases, has demonstrated unprecedented clinical activity in PDGFRA D842V-mutant gastrointestinal stromal tumors (GIST).

**Methods:**

This retrospective analysis compared efficacy of avapritinib in patients enrolled in the NAVIGATOR phase 1 trial (NCT02508532) with the efficacy of other tyrosine kinase inhibitors (TKIs) in patients with unresectable/metastatic PDGFRA D842V-mutant GIST enrolled in a retrospective natural history study (Study 1002). The primary endpoint was overall survival (OS) from the start of reference treatment (avapritinib for NAVIGATOR patients or first-line TKI for treatment of unresectable/metastatic GIST for Study 1002 patients); the secondary endpoint was progression-free survival (PFS). Adjusted Kaplan–Meier survival curves were compared by Cox regression.

**Results:**

Fifty-six (NAVIGATOR) and 19 (Study 1002) patients with PDGFRA D842V-mutant GIST were evaluated; of the 56 patients from NAVIGATOR, a subgroup of patients treated with either 300 mg (recommended phase 2 dose) or 400 mg (maximum tolerated dose) avapritinib starting dose (*n* = 38) were analyzed separately. Patient characteristics were adjusted for imbalances by propensity score between the study groups. Inverse probability of treatment weighting-adjusted Kaplan–Meier analysis of OS showed median OS was not reached for NAVIGATOR patients treated with any of the avapritinib doses tested and was 12.6 months for Study 1002 patients; OS rate at 6/48 months was 100%/63% in NAVIGATOR and 56%/17% in Study 1002 (*P =* 0.0001). In the 300/400 mg subgroup, adjusted OS rates at 6/36 months were 100%/73 and 68%/20% in Study 1002 (*P =* 0.0016). Adjusted median PFS was 29.5 months in NAVIGATOR and 3.4 months in Study 1002.

**Conclusions:**

In this indirect, retrospective analysis, avapritinib demonstrated more durable survival outcomes compared with other TKIs in patients with unresectable/metastatic PDGFRA D842V-mutant GIST.

**Trial registration:**

The NAVIGATOR trial was registered at ClinicalTrials.gov as per July 2015, Identifier: NCT02508532.

**Supplementary Information:**

The online version contains supplementary material available at 10.1186/s12885-021-08013-1.

## Background

Over 85% of gastrointestinal stromal tumors (GIST) are driven by oncogenic mutations of the genes encoding KIT and/or platelet-derived growth factor receptor A (PDGFRA) receptor tyrosine kinases [1, 2]. The most common sites for mutations in GIST are in the juxtamembrane domain (exon 11; 60–70%) and the extracellular domain (exon 9; 5–10%) of *KIT*; mutations in *PDGFRA* (5–10%) are most commonly located in the activation loop (exon 18) and the juxtamembrane domain (exon 12) [3–6].

Tyrosine kinase inhibitors (TKIs), developed to target pathogenic mutant kinases, have revolutionized the treatment landscape for patients with unresectable or metastatic GIST over the past two decades [7]. US and European treatment guidelines for GIST strongly recommend genetic testing for *KIT* and *PDGFRA* mutations, due to their response-predictive value and thus significance in guiding treatment decisions [8–10]. However, patients with unresectable/metastatic PDGFRA D842V-mutant GIST have a poor prognosis because imatinib and other approved TKIs lack activity against PDGFRA D842V-mutant kinases [3, 11, 12]. Approved treatments have provided, at best, very few objective responses in patients with the D842V mutation in clinical trials [13, 14]. For example, published studies have shown very infrequent responses with imatinib in PDGFRA D842V-mutant GIST, with only 3% (two out of 59) of patients across studies achieving a partial response to this treatment [3, 13–16]. To date, median progression-free survival (PFS) is between 2 and 10 months and median overall survival (OS) is approximately 9–25 months [13, 14, 17], thereby highlighting the urgent unmet medical need for patients with unresectable/metastatic GIST harboring the *PDGFRA* D842V mutation.

Avapritinib (formerly BLU-285, Blueprint Medicines Corporation, Cambridge, Massachusetts, USA) is a selective, potent inhibitor of KIT and PDGFRA mutant kinases, which is currently approved in the US for the treatment of adults with unresectable or metastatic GIST that harbor a *PDGFRA* exon 18 mutation, including D842V [18]. Avapritinib has also been approved in the EU for the treatment of adult patients with unresectable or metastatic GIST harboring the *PDGFRA* D842V mutation [19]. These approvals were based on the open-label, non-randomized, phase 1, dose escalation and dose expansion NAVIGATOR (ClinicalTrials.gov: NCT02508532) trial, designed to evaluate the safety and antineoplastic activity of avapritinib in patients with unresectable/metastatic GIST, previously treated with TKIs. In this study, avapritinib showed unprecedented clinical efficacy and durable responses in patients with unresectable or metastatic PDGFRA D842V-mutant GIST. Study 1002 was a retrospective, observational, chart-based natural history study that evaluated the response and survival of patients with unresectable/metastatic PDGFRA D842V-mutant GIST treated during their clinical course with TKIs other than avapritinib [20]. The objective of this analysis was to retrospectively compare efficacy outcomes in patients treated with avapritinib in the NAVIGATOR trial with patients treated with other TKIs in Study 1002.

## Methods

### Study design and patients

Patients with unresectable/metastatic GIST harboring a *PDGFRA* D842V mutation were enrolled in NAVIGATOR, or retrospectively selected for Study 1002, based on their treatment history. For this analysis, the data cut-off for the NAVIGATOR trial was March 9, 2020. The detailed study design for NAVIGATOR was described previously [21]. Briefly, patients were enrolled in NAVIGATOR if they were ≥ 18 years of age with a histologically- or cytologically-confirmed diagnosis of unresectable GIST that had progressed following imatinib and ≥ 1 of the following: sunitinib, regorafenib, sorafenib, dasatinib, pazopanib or an experimental kinase-inhibitor agent, or had a disease with a D842V mutation in the *PDGFRA* gene. Mutational status was determined by local testing and centrally confirmed using circulating tumor DNA as well as archival or new tumor biopsy samples.

In Study 1002, which was conducted at three US sites, patients ≥ 18 years of age with a confirmed diagnosis of GIST harboring a D842V mutation in PDGFRA were treated with a commercially available or investigational TKI. Patients were excluded from Study 1002 if they were enrolled in a clinical trial of avapritinib or if they had received prior TKI therapy only in an adjuvant setting. Demographic and clinical data from patients following each line of therapy were gathered retrospectively between January 2000 and July 2016.

### Study endpoints

The primary endpoint of the current analysis was OS and the secondary endpoint was PFS, assessed in Study 1002 by Response Evaluation Criteria in Solid Tumors version 1.1 [22] and in NAVIGATOR by Response Evaluation Criteria in Solid Tumors version 1.1 with modifications for GIST as previously defined and used in a phase 3 trial of regorafenib [23]. OS and PFS were measured either from the start of treatment with avapritinib (in NAVIGATOR) or the first TKI prescribed for unresectable/metastatic GIST (in Study 1002), to the date of death event (OS and PFS) or disease progression (PFS).

### Statistical analysis

The Kaplan–Meier method was used to obtain inverse probability of treatment weighting (IPTW)-adjusted and unadjusted survival curves for patients treated with avapritinib (NAVIGATOR) and other commercially available or investigational TKIs (Study 1002). IPTW-adjusted Kaplan–Meier survival functions were estimated using calculated propensity score (PS) weighting, with the PS used to adjust for imbalances in the characteristics of patients included in NAVIGATOR and Study 1002. Calculated weights were generated using a PS multivariate logistic regression model that included age and presence of metastatic disease at the start of reference treatment, anatomical site of primary tumor at diagnosis, duration of disease from diagnosis to start of reference treatment, sex, and number of TKIs (counted from the first TKI for treatment of unresectable/metastatic GIST). Race and Eastern Cooperative Oncology Group (ECOG) performance status variables were not included due to a relatively high number of missing values. Each patient was assigned an individual weight based on the PS regression, with the weighted mean of the characteristics harmonized between the cohorts. With the IPTW method, patients in Study 1002 (control group) with a very high similarity to the baseline characteristics of the avapritinib treatment group (NAVIGATOR) are given relatively higher weights, whereas those with very high propensity scores will have lower weights. Log-rank and Cox regression-based tests were used to compare unadjusted and adjusted Kaplan–Meier survival curves, respectively, with the aim of testing the null hypothesis that there was no difference between survival curves for NAVIGATOR and Study 1002. All analyses were conducted using STATA software (Release 13; StataCorp, College Station, Texas, USA).

## Results

### Patients

**Fig. 1:**
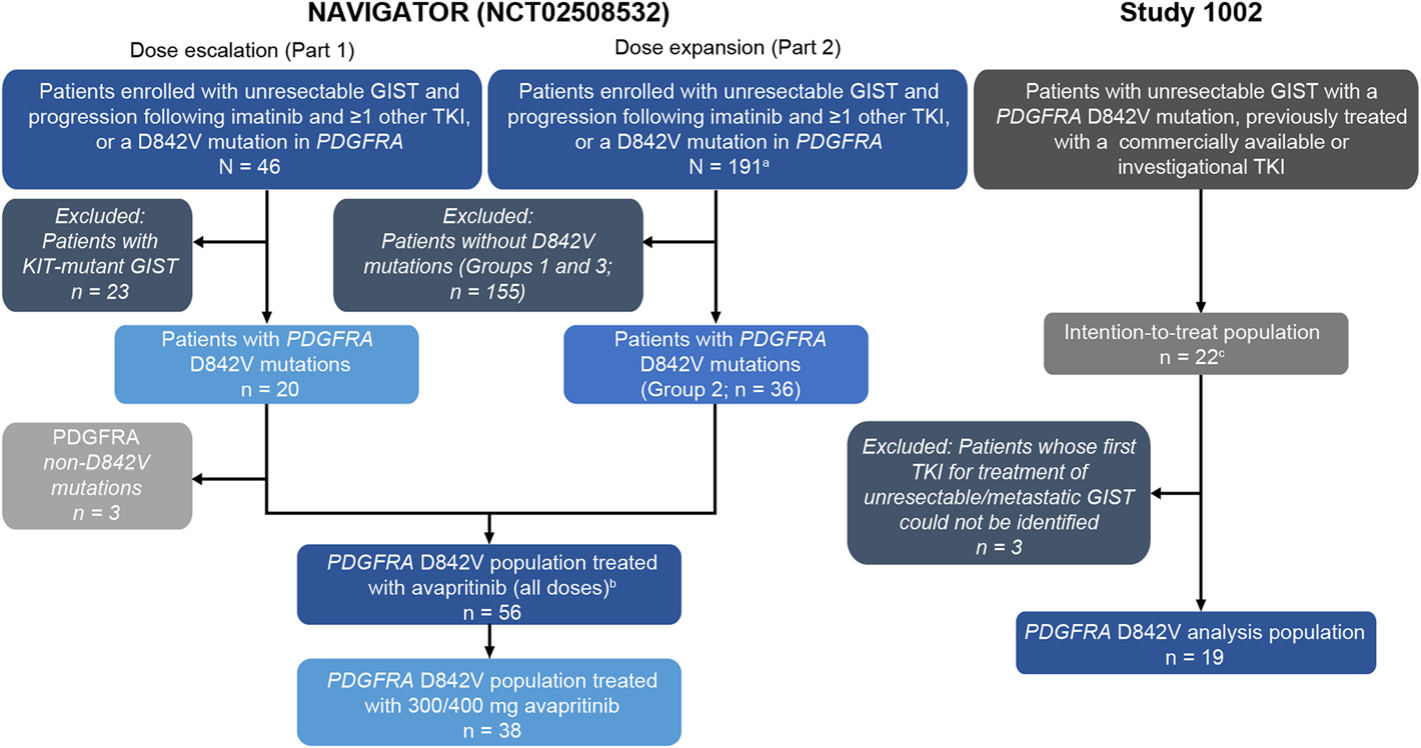
Patient disposition. Abbreviations: GIST, gastrointestinal stromal tumor; *PDGFRA*, platelet-derived growth factor receptor A; TKI, tyrosine kinase inhibitor. ^a^Enrollment at the data cutoff of November 16, 2018. ^b^The D842V population included all patients with *PDGFRA* D842V mutations from part 1 and part 2. ^c^All patients were treated with an approved treatment for GIST or investigational TKI, with initial treatment administered between January 1, 2000, and July 1, 2016

A total of 56 patients in NAVIGATOR (all patients with PDGFRA D842V-mutant GIST) and 19 patients in Study 1002 were included in the analysis; of the 56 patients in NAVIGATOR, a subgroup of 38 patients who started avapritinib treatment at 300 mg or 400 mg was also analyzed separately (Fig. 1). In NAVIGATOR, the maximum tolerated dose was determined as 400 mg and the recommended phase 2 dose was 300 mg [21]. Although 22 patients were included in Study 1002 in total, three were excluded from the current analysis as the first TKI treatment for unresectable/metastatic GIST could not be identified (these patients only received imatinib in the adjuvant setting). Data were restricted to variables that were available in both studies at comparable time points (i.e., at the start of reference treatment).

Overall (*N* = 75; 56 patients in NAVIGATOR and 19 patients in Study 1002), the majority of patients were male (*n* = 51; 68%), had GIST located in the stomach (*n* = 61; 81%), and over half the patients (*n* = 45; 60%) had unresectable/metastatic GIST for < 3 years. Patient characteristics were balanced between the two studies for sex, race, tumor anatomical location, presence of metastatic disease, and duration of disease (Table 1). An analysis using the chi-square test showed that age was the only patient characteristic that was significantly different between the two cohorts (*P =* 0.046); in NAVIGATOR, 32% (18/56) of patients were younger than 60 years, compared with 58% (11/19) of patients in Study 1002. Overall, patients enrolled in NAVIGATOR received fewer previous lines of TKIs, with 61% (34/56) of patients having received ≤ 2 prior lines of TKIs compared with only 32% (6/19) in Study 1002; the highest proportion of patients had received two prior lines of TKIs (23/56; 41%) in NAVIGATOR compared with ≥ 4 prior lines of TKIs (9/19; 47%) in Study 1002. Pre-avapritinib treatments received by patients in NAVIGATOR and treatments received by patients in Study 1002 are presented in Supplementary Tables 1 and 2, respectively. In NAVIGATOR and Study 1002, the most commonly prescribed first- and second-line treatments were imatinib (NAVIGATOR: 36/42 [86%]; Study 1002: 20/22 [91%]) and sunitinib (NAVIGATOR: 14/24 [58%]; Study 1002: 9/19 [47%]), respectively. One patient received docetaxel/gemcitabine as a second-line treatment in Study 1002. There was no clear treatment preference among third- or fourth-line agents. In NAVIGATOR, one patient received paclitaxel as a third-line treatment.

**Table 1:** Analysis of confounding patient-related factors

Patient characteristic, n (%)	NAVIGATOR		Study 1002 *n* = 19	*P* value	
	All doses ***n*** = 56	300/400 mg ***n*** = 38		NAVIGATOR All doses vs Study 1002	NAVIGATOR 300/400 mg vs Study 1002
Sex				0.601	0.844
Male	39 (70)	25 (66)	12 (63)		
Female	17 (30)	13 (34)	7 (37)		
Age^a^				0.046*	0.088
< 60 years	18 (32)	13 (34)	11 (58)		
≥ 60 years	38 (68)	25 (66)	8 (42)		
Race				0.101	0.042*
White	39 (78)	25 (71)	18 (95)		
Non-white	11 (22)	10 (29)	1 (5)		
Missing	6	3	0		
Anatomical site^b^				0.757	0.823
Gastric (stomach)	46 (82)	29 (76)	15 (79)		
Small bowel or rectal (any other organ)	10 (18)	9 (24)	4 (21)		
Metastatic disease^a^				0.745	0.611
No	2 (4)	1 (3)	1 (5)		
Yes	54 (96)	37 (97)	18 (95)		
ECOG Performance Status^a^				0.445	0.400
0	21 (38)	13 (34)	1 (100)		
1	32 (57)	23 (61)	0		
≥ 2	3 (5)	2 (5)	0		
Missing	0	0	18		
Duration of disease^c^				0.386	0.340
< 3 years	32 (57)	21 (55)	13 (68)		
≥ 3 years	24 (43)	17 (45)	6 (32)		
Number of TKI treatment lines^d^				0.124	0.040*
1	11 (20)	5 (13)	3 (16)		
2	23 (41)	20 (53)	3 (16)		
3	9 (16)	6 (16)	4 (21)		
≥ 4	13 (23)	7 (18)	9 (47)		

Abbreviations: ECOG*,* Eastern Cooperative Oncology Group; *TKI,* tyrosine kinase inhibitor

Not all available baseline and demographic characteristics could be used due to differences in the timing of the measure (i.e., screening in NAVIGATOR vs diagnosis in Study 1002)

^a^Estimated at the start of reference treatment

^b^Recorded at the primary diagnosis

^c^Estimated from the date of diagnosis to the date of start of reference treatment

^d^The number of lines of TKIs was counted from the first TKI for treatment of unresectable/metastatic disease including avapritinib for NAVIGATOR population

*P value statistically significant (≤ 0.05); comparison between NAVIGATOR population vs Study 1002 population

In the 300/400 mg subgroup (*n* = 38), patient characteristics were also well balanced when compared with Study 1002. Chi-square tests showed patient race and the total number of prior lines of TKIs were the only significantly different factors (*P =* 0.042 and *P =* 0.040, respectively; Table 1). In the NAVIGATOR 300/400 mg subgroup, a lower proportion of patients were white (71%; 25/35) compared with patients in Study 1002 (95%; 18/19). In NAVIGATOR, similar to the overall population, patients in the 300/400 mg subgroup received fewer previous lines of TKI therapy than patients in Study 1002.

### Overall survival

**Fig. 2:**
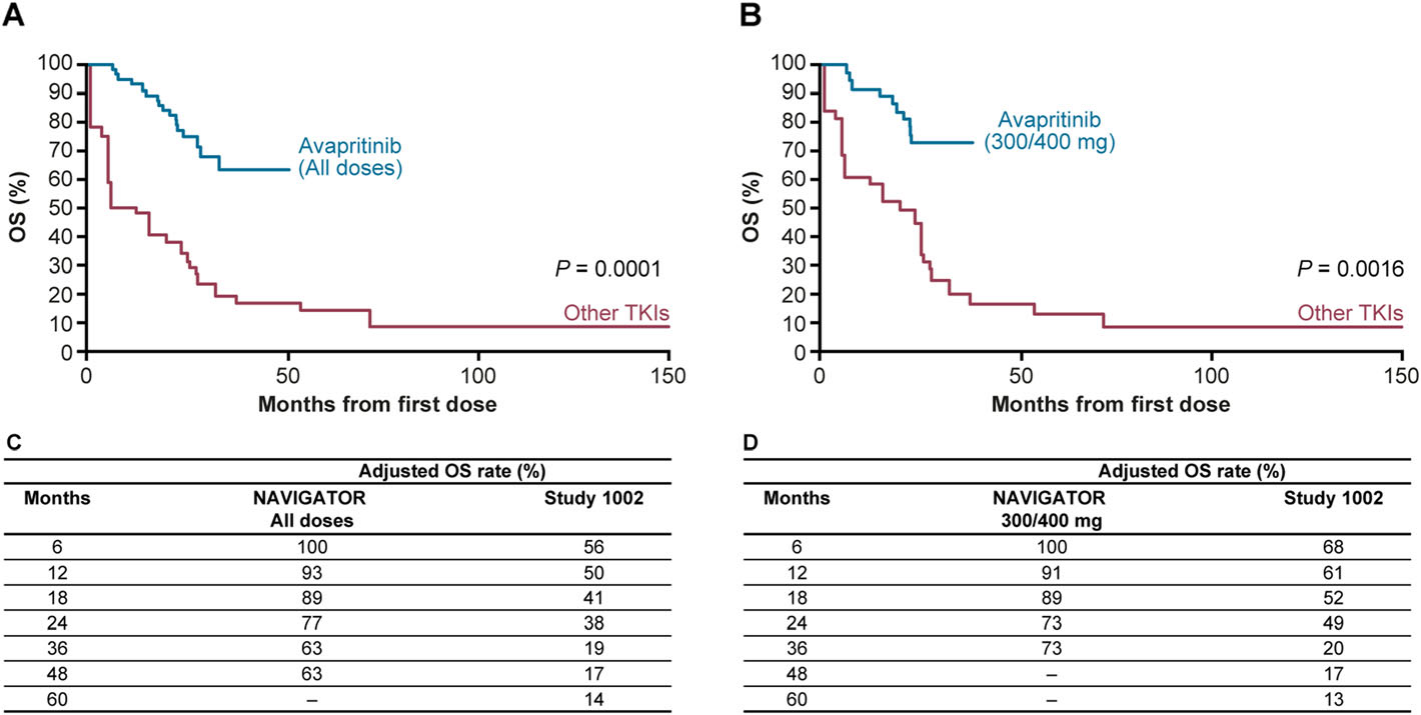
Adjusted Kaplan–Meier overall survival estimates. **a** Overall NAVIGATOR population vs Study 1002. **b** NAVIGATOR 300/400 mg subgroup vs Study 1002. **c** Adjusted OS landmark analyses for NAVIGATOR population and Study 1002. **d** Adjusted OS landmark analyses for NAVIGATOR 300/400 mg subgroup and Study 1002

**Fig. 3:**
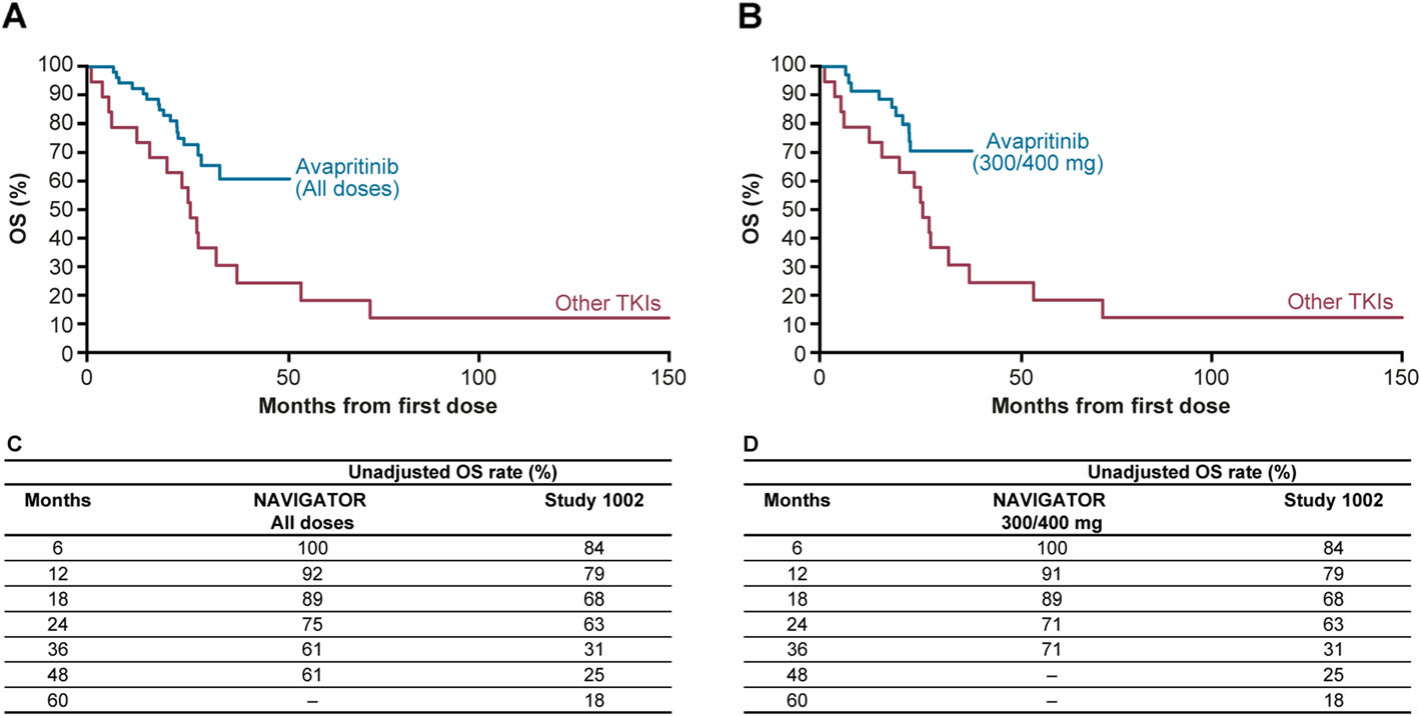
Unadjusted Kaplan–Meier overall survival estimates. **a** Overall NAVIGATOR population vs Study 1002. **b** NAVIGATOR 300/400 mg subgroup vs Study 1002. **c** Undjusted OS landmark analyses for NAVIGATOR population and Study 1002. **d** Unadjusted OS landmark analyses for NAVIGATOR 300/400 mg subgroup and Study 1002

Median OS was not reached in NAVIGATOR in both the adjusted and unadjusted analyses while in Study 1002, median OS was 12.6 months and 26.4 months, respectively. The Kaplan–Meier estimates for OS for NAVIGATOR (all patients) and the 300/400 mg subgroup compared with Study 1002 are presented in Fig. 2 (adjusted) and Fig. 3 (unadjusted), respectively. Adjusted and unadjusted OS rates were greater in the overall NAVIGATOR population (Figs. 2c and 3c) and in the 300/400 mg subgroup compared with Study 1002 (Figs. 2d and 3d) at all landmarks through to 24 and 36 months. In the adjusted analysis, OS rate at 48 months was 63% in the overall NAVIGATOR population compared with 17% for Study 1002. In the 300/400 mg subgroup, adjusted OS rate at 36 months was 73% compared with 20% in Study 1002.

Based on Cox regression analysis, the null hypothesis was rejected for the adjusted analysis for overall NAVIGATOR population (*P =* 0.0001) and the 300/400 mg subgroup (*P =* 0.0016), demonstrating that the difference between the survival curves, which favored avapritinib compared with other TKIs, was statistically significant.

### Progression-free survival

**Fig. 4:**
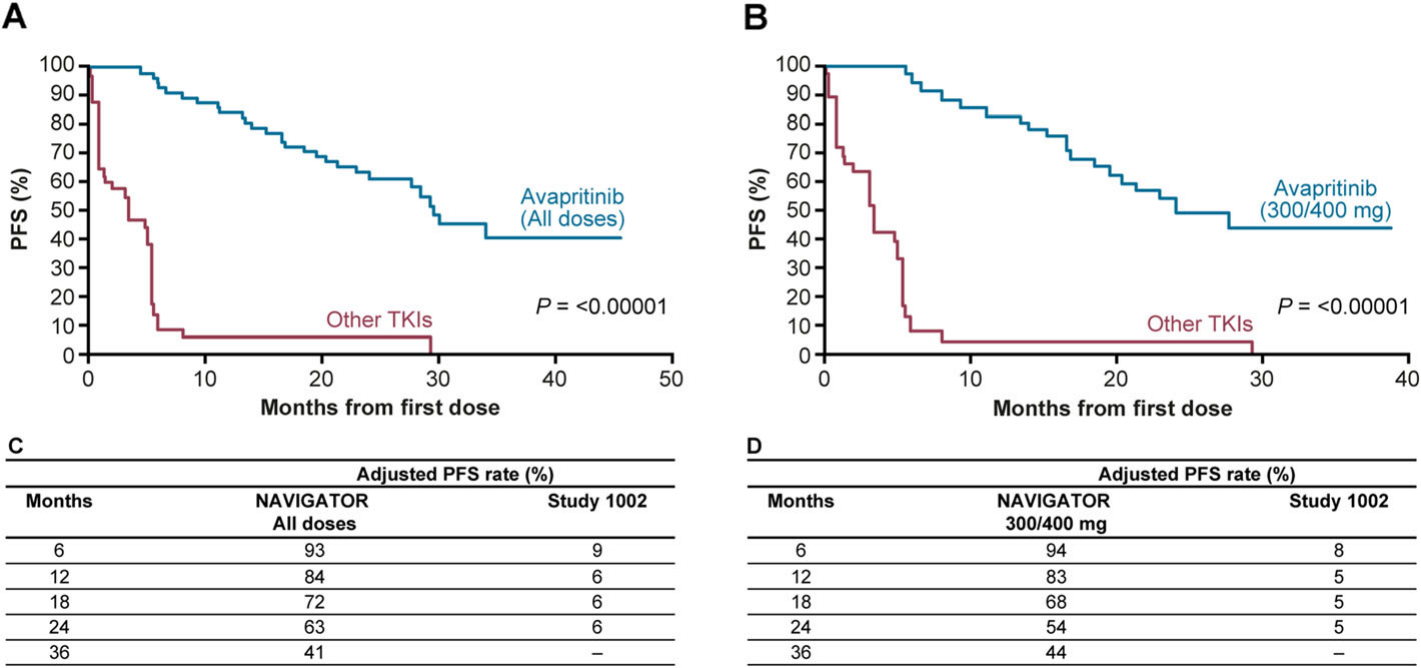
Adjusted Kaplan–Meier progression-free survival estimates. **a** Overall NAVIGATOR population vs Study 1002. **b** NAVIGATOR 300/400 mg subgroup vs Study 1002. **c** Adjusted PFS landmark analyses for NAVIGATOR population and Study 1002. **d** Adjusted PFS analyses for NAVIGATOR 300/400 mg subgroup and Study 1002

**Fig. 5:**
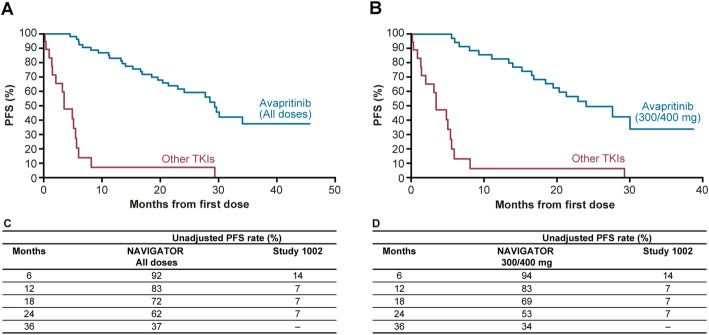
Unadjusted Kaplan–Meier progression-free survival estimates. **a** Overall NAVIGATOR population vs Study 1002. **b** NAVIGATOR 300/400 mg subgroup vs Study 1002. **c** Unadjusted PFS landmark analyses for NAVIGATOR population and Study 1002. **d** Unadjusted PFS analyses for NAVIGATOR 300/400 mg subgroup and Study 1002

Median PFS was 29.5 months in the adjusted and 29.2 months in the unadjusted analysis in NAVIGATOR compared with 3.4 months in Study 1002 (both adjusted and unadjusted). For PFS, the Kaplan–Meier estimates are shown in Fig. 4 (adjusted) and Fig. 5 (unadjusted). Adjusted and unadjusted PFS rates were greater in the overall NAVIGATOR population (Figs. 4c and 5c) and in the 300/400 mg subgroup compared with Study 1002 (Figs. 4d and 5d) at all landmarks through 12 and 24 months. In the adjusted analysis, PFS rate at 24 months was 63% in the overall NAVIGATOR population compared with 6% for Study 1002. In the 300/400 mg subgroup, adjusted PFS rate at 24 months was 54% compared with 5% in Study 1002. In both cohorts of NAVIGATOR (all patients and 300/400 mg subgroup), the null hypothesis was rejected for this analysis as the test was significant (*P <* 0.00001).

## Discussion

This is the first retrospective statistical analysis conducted to evaluate treatment outcomes with avapritinib compared with other TKIs in patients with unresectable/metastatic PDGFRA D842V-mutant GIST. Data in this patient population are currently limited due to the low prevalence of GIST associated with *PDGFRA* D842V mutations. Additionally, very limited data are available on the outcome of treatment with TKIs in these patients.

In the most recently published NAVIGATOR trial results, avapritinib demonstrated significant clinical activity and durable responses in patients with unresectable/metastatic PDGFRA D842V-mutant GIST. In patients treated with 300/400 mg, the centrally confirmed overall response was 95% (36/38 patients, 95% confidence interval [CI] 82–99) with 5 (13%) complete responses and 31 (82%) partial responses. Median duration of response was 22 months (95% CI 14–not reached) and median PFS was 24 months (95% CI 18–not reached); median OS was not reached at a median follow-up of 27.5 months, with an estimated OS rate of 71% at 36 months [24]. In the analysis reported here, statistical adjustments were made to control for confounding factors in order to determine the primary and secondary outcomes. IPTW-adjusted OS and PFS analysis was chosen over more conventional matching methods for several reasons: first, unlike other matching methods study patients are rarely eliminated from the analysis, which is suitable for studies which have a limited sample size and allowed retention of the highest possible number of observations in the analysis; second, IPTW serves to reduce or remove the effects of confounding factors in non-randomized studies; finally, the application of the IPTW method has also been reported in two similar recently published analyses (these consisted of single-arm studies of ceritinib and blinatumomab compared with historical control groups). Findings from both these trials supported regulatory approvals [25, 26]. In Study 1002, the median adjusted and unadjusted OS differed (12.6 months and 26.4 months respectively), whereas the median PFS adjusted and unadjusted values were the same (3.4 months). The greater impact of adjustment on OS compared with PFS may be due to the greater range and variability of OS values. In addition, the smaller number of death events compared with progression events may also be an explanation.

Our indirect analysis has a number of limitations. In Study 1002, OS was measured from the start of the first TKI used for unresectable/metastatic GIST, but in the NAVIGATOR trial, OS was measured from the start of avapritinib treatment. Therefore, the impact of previous TKI therapies on the survival benefits of avapritinib cannot be extrapolated from these results, although all patients included in this study had PDGFRA D842V-mediated resistance to currently approved TKI-based therapies. It is important to note that avapritinib is currently approved for unresectable/metastatic PDGFRA D842V-mutant GIST, regardless of the line of therapy [18, 19]. In real-world clinical practice, avapritinib is expected to be used as a first-line TKI for this indication, hence, it will be crucial to collect and publish data evaluating avapritinib in the first-line setting for unresectable/metastatic PDGFRA D842V-mutant GIST. In addition, although patient characteristics were generally well balanced between the two studies, age (overall NAVIGATOR population), race, and the total number of prior lines of TKI therapy (NAVIGATOR 300/400 mg subgroup) were significantly different compared with patients in Study 1002. The higher number of prior TKI treatments received by patients in Study 1002 patients reflects the use of available TKIs with the characteristic treatment resistance to *PDGFRA* D842V mutations before the approval of avapritinib for PDGFRA exon 18 and D842V-mutant GIST. Furthermore, analyses of retrospective studies such as Study 1002 risk overestimation of PFS duration. However, despite this risk, we still observed significant differences in PFS between NAVIGATOR and Study 1002. Comparisons between PFS may also have been impacted by differences in evaluation criteria between NAVIGATOR and Study 1002; OS comparisons were not affected by differences in assessment criteria. It should also be noted that *P* values should be interpreted with caution as this is an indirect analysis. Lastly, as a retrospective study, adverse event data were not collected for Study 1002, so safety comparisons with NAVIGATOR could not be conducted. A comprehensive post-hoc analysis of the safety and tolerability of avapritinib in the dose escalation/expansion phase I NAVIGATOR study, including guidance on management of cognitive effects and intracranial bleeding events, has been presented previously [27].

## Conclusions

This retrospective, indirect analysis suggests avapritinib leads to more durable survival outcomes in patients with PDGFRA D842V-mutant unresectable/metastatic GIST than other TKIs used for treating patients with GIST. In addition, Study 1002 confirmed that the prognosis of patients in this population treated with TKIs available prior to avapritinib approval was poor. Overall, these results underscore the importance of tumor mutational testing at initial diagnosis, to ensure the most effective and clinically appropriate treatment is provided.

## Supplementary Information

**Additional file 1: Supplementary Table 1.** Prior treatments received by patients in NAVIGATOR. **Supplementary Table 2.** Prior treatments received by patients in Study 1002. **Supplementary Table 3.** NAVIGATOR study investigational sites and institutional review boards (IRB) or independent ethics committees (EC) list. **Supplementary Table 4.** Study 1002 investigational sites and institutional review boards (IRB) list.

## Data Availability

The anonymized derived data from NAVIGATOR and Study 1002 that underlie the results reported in this article will be made available, beginning 12 months and ending 5 years following this article’s publication, to any investigators who sign a data access agreement and provide a methodologically sound proposal to medinfo@blueprintmedicines.com. The trial protocol will also be made available, as will a data fields dictionary.

## Abbreviations

GIST: Gastrointestinal stromal tumors
PDGFRA: Platelet-derived growth factor receptor A
TKIs: Tyrosine kinase inhibitors
PFS: Progression-free survival
OS: Overall survival
iPTW: Inverse probability of treatment weighting
PS: Propensity score
ECOG: Eastern Cooperative Oncology Group
