# Supplementary material for: Clinical efficacy comparison of avapritinib with other tyrosine kinase inhibitors in gastrointestinal stromal tumors with PDGFRA D842V mutation: a retrospective analysis of clinical trial and real-world data
Source: BMC Cancer. 2021 Mar 19;21:291. doi: 10.1186/s12885-021-08013-1 (PMC7976710; doi:10.1186/s12885-021-08013-1)
Supplement: Supplementary file 1 — Additional file 1: Supplementary Table 1. Prior treatments received by patients in NAVIGATOR. Supplementary Table 2. Prior treatments received by patients in Study 1002. Supplementary Table 3. NAVIGATOR study investigational sites and institutional review boards (IRB) or independent ethics committees (EC) list. Supplementary Table 4. Study 1002 investigational sites and institutional review boards (IRB) list. [file 12885_2021_8013_MOESM1_ESM.docx]

# Supplementary information

# Supplementary Table 1 Prior treatments received by patients in NAVIGATOR

| **Prior treatment, n (%)** | **All patients N = 56** |
| --- | --- |
| **First-line therapy** | **42 (75)** |
| Imatinib^a^ | 36 (86) |
| Crenolanib | 3 (7) |
| Regorafenib | 2 (5) |
| Sunitinib | 1 (2) |
| **Second-line therapy** | **24 (43)** |
| Sunitinib | 14 (58) |
| Imatinib | 4 (17) |
| Dasatinib | 2 (8) |
| Crenolanib | 1 (4) |
| Regorafenib | 1 (4) |
| Sorafenib | 1 (4) |
| Sunitinib + sirolimus | 1 (4) |
| **Third-line therapy** | **15 (27)** |
| Sorafenib | 4 (27) |
| Dasatinib | 3 (20) |
| Regorafenib | 3 (20) |
| Crenolanib | 2 (13) |
| Regorafenib + sirolimus | 1 (7) |
| Sunitinib | 1 (7) |
| Paclitaxel | 1 (7) |
| **Fourth-line therapy** | **7 (13)** |
| Regorafenib | 3 (43) |
| Imatinib | 1 (14) |
| Dasatinib | 1 (14) |
| Sorafenib | 1 (14) |
| Sunitinib | 1 (14) |

^a^Four patients were given imatinib as adjuvant therapy and these patients underwent surgery for total tumor resection.

# Supplementary Table 2 Prior treatments received by patients in Study 1002

| **Prior treatment, n (%)** | **All patients (Intention to treat population; N = 22)** |
| --- | --- |
| **First-line therapy** | **22 (100)** |
| Imatinib | 20 (91) |
| Nilotinib | 1 (5) |
| Sunitinib | 1 (5) |
| **Second-line therapy** | **19 (86)** |
| Sunitinib | 9 (47) |
| Imatinib | 4 (21) |
| Investigational agent | 3 (16) |
| Regorafenib | 1 (5) |
| Dasatinib | 1 (5) |
| Docetaxel/gemcitabine | 1 (5) |
| **Third-line therapy** | **16 (73)** |
| Sunitinib | 4 (25) |
| Dasatinib | 4 (25) |
| Imatinib | 4 (25) |
| Investigational agent | 2 (13) |
| Imatinib/gemcitabine | 1 (6) |
| Regorafenib | 1 (6) |

# Supplementary Table 3 NAVIGATOR study investigational sites and institutional review boards (IRB) or independent ethics committees (EC) list

| **Site** | **IRB/EC** |
| --- | --- |
| Oregon Health and Science University, Portland, OR, USA | Oregon Health and Science University IRB, Portland, OR, USA |
| Royal Marsden Hospital, London, UK | South Central - Berkshire B Research EC, Bristol, UK |
| Dana-Farber Cancer Institute, Boston, MA, USA | Dana-Farber Cancer Institute IRB, Boston, MA, USA |
| Fox Chase Cancer Center, Philadelphia, PA, USA | Quorum Review IRB, Seattle, WA, USA |
| University of Duisburg-Essen, Essen, Germany | Ethik-Kommission der medizinischen Fakultät der Universität Duisburg-Essen, Essen, Germany |
| Asan Medical Center, Seoul, South Korea | Asan Medical Center IRB, Seoul, South Korea |
| Leuven Cancer Institute, University Hospitals Leuven, Leuven, Belgium | UZ Leuven EC, Leuven, Belgium |
| Institut Gustave Roussy, Gustave Roussy Cancer Campus, Villejuif, France | CPP SUD EST IV, Lyon, France |
| Erasmus MC Cancer Institute, Rotterdam, The Netherlands | Medische Ethische Toetsings  Commissie, Rotterdam, The Netherlands |
| Vall d' Hebron Institute of Oncology, Barcelona, Spain | CEIC del Hospital Universitari Vall d'Hebron, Barcelona, Spain |
| Centre Léon Bérard, Lyon, France | CPP SUD EST IV, EC, Lyon, France |
| Memorial Sloan Kettering Cancer Center, New York, NY, USA | Memorial Sloan Kettering Cancer Center IRB, New York, NY, USA |
| Sylvester Comprehensive Cancer Center, Miami, FL, USA | University of Miami IRB, Miami, FL, USA |
| Klinika Nowotworów Tkanek Miękkich, Maria Sklodowska-Curie National Research Institute of Oncology, Warsaw, Poland | Komisji Bioetycznej przy Centrum Onkologii -Instytucie im. Marii Skłodowskiej-Curie, Warsaw, Poland |
| MD Anderson, Houston, TX, USA | MD Anderson Cancer Center IRB, Houston, TX, USA |
| Sarcoma Oncology Center, Santa Monica, CA, USA | Western IRB, Puyallup, WA, USA |
| Cancer Treatment Centers of America, Newnan, GA, USA | Western IRB, Puyallup, WA, USA |

# Supplementary Table 4 Study 1002 investigational sites and institutional review boards (IRB) list

| **Site** | **IRB** |
| --- | --- |
| Oregon Health and Science University, Portland, OR, USA | Oregon Health and Science University IRB, Portland, OR, USA |
| Dana-Farber Cancer Institute, Boston, MA, USA | Dana-Farber Cancer Institute IRB, Boston, MA, USA |
| Fox Chase Cancer Center, Philadelphia, PA, USA | Fox Chase Cancer Center IRB, Rockledge, PA, USA |
